# Retraction: SARS-CoV2 mRNA vaccine intravenous administration induces myocarditis in chronic inflammation

**DOI:** 10.1371/journal.pone.0349831

**Published:** 2026-05-21

**Authors:** The PLOS One Editors

Following the publication of this article [1], concerns were raised regarding similarity to another published article [2]. During editorial follow-up, corresponding author BKL stated that they identified critical errors in the management of the histopathology results presented in this article [1] that undermine the validity of the reported findings, and requested retraction of this article.

In light of the above concerns that question the reliability and validity of the published results, the *PLOS One* Editors retract this article [1].

HEJ, SL, JL, GR, HKK, JHS, YJL, COG, JHN, and BKL agreed with the retraction. HJP, YSL, YJK, and ESJ either did not respond directly or could not be reached.
